# Budding pouches and associated bubbles: 3D visualization of exo-membrane structures in *plasmodium falciparum* gametocytes

**DOI:** 10.3389/fcimb.2022.962495

**Published:** 2022-08-22

**Authors:** Eri Saki H. Hayakawa, Marina Wayama, Fuyuki Tokumasu, Nobuhiko Ohno, Mami Matsumoto, Jiro Usukura

**Affiliations:** ^1^ Division of Medical Zoology, Department of Infection and Immunity, Jichi Medical University, Tochigi, Japan; ^2^ Nanostructure Characterization Group, Solution Development Department, Hitachi High-Tech Corporation, Tokyo, Japan; ^3^ Department of Cellular Architecture Studies, Institute of Tropical Medicine (NEKKEN), Nagasaki University, Nagasaki, Japan; ^4^ Division of Histology and Cell Biology, Department of Anatomy, Jichi Medical University, Tochigi, Japan; ^5^ Division of Ultrastructural Research, National Institute for Physiological Sciences, Okazaki, Japan; ^6^ Section of Electron Microscopy, Supportive Center for Brain Research, National Institute for Physiological Sciences, Okazaki, Japan; ^7^ Department of Developmental and Regenerative Neurobiology, Institute of Brain Science, Nagoya City University Graduate School of Medical Sciences, Nagoya, Japan; ^8^ Institute of Material and Systems for Sustainability, Nagoya University, Nagoya, Japan

**Keywords:** *Plasmodium falciparum*, gametocyte, pouch, bubble, large membrane whorls, unroofing-transmission electron microscopy, serial block face scanning electron microscopy, Garnham body

## Abstract

*Plasmodium falciparum* gametocytes have unique morphology, metabolism, and protein expression profiles in their asexual stages of development. In addition to the striking changes in their appearance, a wide variety of “exo-membrane structures” are newly formed in the gametocyte stage. Little is known about their function, localization, or three-dimensional structural information, and only some structural data, typically two-dimensional, have been reported using conventional electron microscopy or fluorescence microscopy. For better visualization of intracellular organelle and exo-membrane structures, we previously established an unroofing technique to directly observe Maurer’s clefts (MCs) in asexual parasitized erythrocytes by removing the top part of the cell’s membrane followed by transmission electron microscopy. We found that MCs have numerous tethers connecting themselves to the host erythrocyte membrane skeletons. In this study, we investigated the intracellular structures of gametocytes using unroofing-TEM, Serial Block Face scanning electron microscopy, and fluorescence microscopy to unveil the exo-membrane structures in gametocytes. Our data showed “balloon/pouch”-like objects budding from the parasitophorous vacuole membrane (PVM) in gametocytes, and some balloons included multiple layers of other balloons. Furthermore, numerous bubbles appeared on the inner surface of the erythrocyte membrane or PVM; these were similar to MC-like membranes but were smaller than asexual MCs. Our study demonstrated *P. falciparum* reforms exo-membranes in erythrocytes to meet stage-specific biological activities during their sexual development.

## Introduction

The gametocytes of *Plasmodium falciparum*, which are the sexual stages of the parasite found in human erythrocytes, undergo a series of developmental stages (I to V). During gametocytogenesis, the parasites significantly change their morphology from round in shape in the early stages to the typical “banana” shape of mature gametocytes (Dixon et al., 2012). These structural changes in infected erythrocytes are associated with alterations to the spectrin network of the parasite cell membrane in stages III–V (Dearnley et al., 2016) and rearrangements of F-actin and tubulin localization (Hliscs et al., 2015). The cell-surface protrusions, called “knobs”, that can be observed on asexual parasitized erythrocytes disappear in cells containing the gametocytes (Tiburcio et al., 2013). Furthermore, skeleton-binding protein-1, a marker protein for the Maurer’s cleft (MC), which is parasitized-cell-specific membrane structure responsible for protein transport in asexual stages, showed continuous fluorescence along the edge of infected cells instead of the discontinuous patterns detected in asexual stages (Kats et al., 2015). Other studies also suggested the presence of complex exo-membrane structures in the gametocyte-infected erythrocyte cytoplasm and possible extensions of parasitophorous vacuole membranes. These observations suggest that significant re-constructions and/or modifications of the parasite-derived membrane system occur during gametocytogenesis for optimizing gametocyte-specific protein expression and transport (to the erythrocyte surface) (Neveu and Lavazec, 2019). Modifications of parasite structures and exo-membrane systems are also associated with gametocyte-specific alterations in lipid metabolism (Gulati et al., 2015; Tran et al., 2016; Tanaka et al., 2019), suggesting that gametocytes may adjust their metabolic state following active events, including gamete formation and ex-flagellation.

In our previous study, we applied unroofing-TEM to directly image inside infected cells by opening up the infected cell membrane (Morone et al., 2006; Hayakawa et al., 2015; Morone et al., 2020). Our report revealed the three dimensional (3D)-structure of MCs and found that MCs in matured parasitized erythrocytes are immobilized to the cytoskeleton of the inner side of the erythrocyte membrane, MCs exist as monomers or a complex with other MCs, tethers and strings extend from the MC bodies, and bubble-like membranes bud from MC bodies. This imaging technology has the advantage of preserving intact structures of organelles throughout conventional transmission electron microscopy (TEM), which usually causes ultrastructure loses of ~50 nm or less due to the thickness of diamond knives. By contrast, the “unroofing” technique only removes the upper membrane/layers of erythrocytes, leaving the remaining organelles and other fine structures intact.

In this study, we combined unroofing and stereoscopic tilt imaging techniques to facilitate direct observations of the inner structure of gametocyte-parasitized cells. Serial Block-Face SEM (SBF-SEM) was also used to gather sequential cross-sectional images and re-construct the whole gametocyte structure (Sui et al., 2019; Thai et al., 2019). Our multi-imaging techniques successfully identified unique, but complex, membranous structures inside gametocytes that do not exist in the asexual stages of the parasites.

## Materials and methods

### *P. falciparum* cultivation and gametocyte development

*P. falciparum* (line 3D7) was cultivated as previously described (Tanaka et al., 2019; Hayakawa et al., 2020). Briefly, erythrocytes were washed twice with incomplete RPMI-1640 medium (Invitrogen Life Technologies, Grand Island, NY, USA) supplemented with 50 mg/L hypoxanthine (Sigma, St. Louis, MO, USA), 25 μmol/L sodium bicarbonate (Invitrogen Life Technologies), 25 mmol/L HEPES (Sigma), and 10 μg/mL gentamicin (Invitrogen Life Technologies). The parasites cultures were maintained at 5% hematocrit (Ht) in incomplete RPMI medium supplemented with 5 mg/mL AlbuMAX II Lipid-Rich BSA (Invitrogen Life Technologies) under a 90% N_2_/5% CO_2_/5% O_2_ gas mixture at 37°C. For gametocytogenesis, the NF54::DiCre strain was used because of its high gametocyte-producing ability (Tiburcio et al., 2019). A previously described gametocytogenesis protocol (Tanaka et al., 2019) was followed: 6% Ht with 0.1% parasitemia (day 1) in RPMI supplemented with human plasma and maintained at 3% Ht from day 3. Parasites in the asexual stages were eliminated by adding 50 mM N-acetylglucosamine (Sigma) from day 9 to day 11. The cells were enriched by magnetic column with MACS Separator LS columns (Miltenyi Biotec K.K., Bergisch Gladbach, Germany) on day 12 (Ribaut et al., 2008; Tanaka and Williamson, 2011).

### Fluorescence microscopy

To examine the internal lipid membrane structures in the gametocytes, we used HCS LipidTOX (Invitrogen Life Technologies) (ex/em = 577/609) for neutral lipid staining and LipiORDER (Funakoshi Co., Ltd., Tokyo, Japan) (ex/em = 404/470–550) for membrane staining. LipidTOX data were obtained using Leica SP5 confocal microscope (Leica Microsystems, Bannockburn, IL, USA) with a 63 x objective, and LipiORDER images were collected with a Leica DMI8 epifluorescence microscope with a 100 x objective.

### Sample preparation for SBF-SEM

The procedure for preparing specimens for SBF-SEM was modified from that used in our previous study (Thai et al., 2019). Purified gametocytes were fixed with 2% glutaraldehyde (GA)/0.1 M HEPES and stored at 4°C. The next day, the gametocyte samples were washed with 0.1 M HEPES three times and mixed with 2% agarose (low gelling temperature; Sigma) and 0.1 M PBS (1:1, vol/vol) at 50°C. Each sample (gametocyte pellet + agarose) was mixed with ≈600 µL of pre-mixed 1 mL 4% osmium solution (Nisshin EM Co., Ltd., Tokyo, Japan) and 1 mL 3% KFeCN solution (Nacalai Tesque, Inc., Kyoto, Japan) (1:1, vol/vol) and stored for 1 h at 4°C.

After washing with ultra-pure water three times, samples were treated with 10 mg/mL thiocarbohydrazide (Tokyo Chemical Industry Co., Ltd., Tokyo, Japan) for >20 min at room temperature (RT; ~24–26°C) and washed with water four times (3 min per wash). All samples were fixed in 2% osmium solution/ultra-pure water for 30 min at RT, followed by washing four times with water (3 min per wash) to remove osmium. Samples were then stained with 500–600 µL of 2% uranyl acetate (Electron Microscopy Sciences, Hatfield, PA, USA) and stored overnight at 4°C.

The next day, 500–600 µL of L-glutamate solution with lead nitrate (Nacalai Tesque, Inc.) was added to the samples, which were then incubated for 2 h at 50°C. Next, all samples were dehydrated using serially diluted ethanol (60%, 80%, 90%, 95%, and 100%) and dehydrated acetone. Finally, the samples were infiltrated with Durcupan resin (Sigma), and the resin was cured by incubating at 50°C overnight, 60°C for 12 h, and 70°C overnight.

### SBF-SEM observation and data analysis

Each sample was mounted on an aluminum rivet and trimmed down to sizes smaller than 0.5 mm × 0.5 mm. After gold sputtering with Eiko IB-3 (EIKO Corporation, Tokyo, Japan), the samples were imaged with Sigma (Carl Zeiss, Munich, Germany) equipped with the 3View system (Gatan, Pleasanton, CA, USA). The serial images were acquired at a Z thickness of 50 nm and observed using Fiji/ImageJ. After the structures of interest were marked using Microscopy Image Browser (http://mib.helsinki.fi/), 3D images of the gametocytes were constructed using Amira (FEI Technologies Inc., Hillsboro, OR, USA).

### Preparation of unroofed erythrocytes and freeze-etched replicas and TEM

The procedure for preparing unroofed specimens was modified from that used in our previous studies (Morone et al., 2006; Hayakawa et al., 2015; Morone et al., 2020). Briefly, gametocytes separated using MACS columns were washed twice with incomplete RPMI and collected by centrifugation at 1500 × *g* for 5 min (**Figure 4**). Sapphire micro cover glasses (2.5 mm × 2.5 mm, Matsunami Glass Industries, Ltd, Osaka, Japan) were cleaned, and the hydrophilicity was improved *via* plasma treatment. Next, the glasses were coated with a 1% Alcian blue solution (Hayakawa et al., 2015) and washed with ultra-pure water. Approximately 2 µL of gametocyte pellets was sandwiched between the coated glass, and the top glass was pulled off to unroof the cells (Morone et al., 2020). To avoid alterations of protein and lipid membrane structures, we used KHMgE “inside buffer” during the unroofing process (Hayakawa et al., 2015).

The glass slips containing the unroofed gametocyte membranes were lightly fixed and stored in 2.5% GA (Electron Microscopy Science, Hatfield, PA, USA) at 4°C until used. All specimens were washed twice with KHMgE “inside buffer” (Hayakawa et al., 2015) and twice with ultra-pure water, followed by submersion in a 5% ethanol solution for 15 min. After washing with water, the unroofed specimens were frozen in liquid nitrogen using cooled-copper blocks in an RF 23 rapid freezing device (Eiko Engineering Co. Ltd., Mito, Japan). After platinum (Pt) and carbon (C) etching processes for the specimen, the replica samples were separated from sapphire cover glass in 10% hydrogen fluoride solution. Each floating replica was then scooped by 200 thin bar copper grids (Agar Scientific Limited, Essex, England). All unroofed specimens were analyzed using TEM (Hitachi H-7650, Hitachi High-tech, Tokyo, Japan), as described in a previous study (Hayakawa et al., 2015).

### Observation of unroofing specimens by tilting TEM

Tilting images of unroofing specimens were modulated as performed in a previous study (Morone et al., 2006). The tilt angles were changed every 2° intervals from −60° to +60° using HT 7800 TEM (Hitachi High-tech, Tokyo, Japan). Corrections of all images and take alignment were performed by EMIP-EX software with HT 7800 TEM (Hitachi High-tech).

## Results

**Figure 1:**
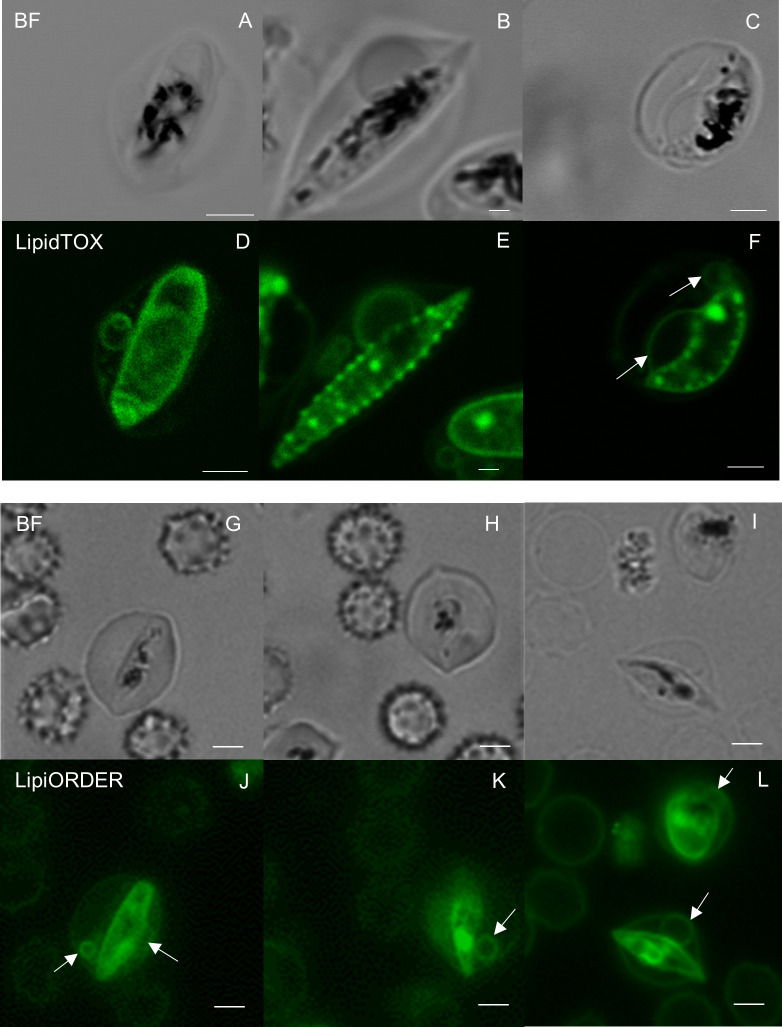
Fluorescence microscopy analyses of gametocyte membranes **(A–C, G–I)** Bright field and **(D–F)** neutral lipid staining images of gametocytes show multiple annular-neutral lipids in the cytosol of *Plasmodium falciparum*–infected erythrocytes. The largest size of circular membranous structures was approximately 4 µm in **(F)**. Membrane lipid staining images also indicate the presence of similar structures by LipiORDER staining **(J–L)**. The white arrows show lipid membranes. Scale bar = 2.5 µm.

We first investigated the internal structures of gametocytes using two fluorescence probes, LipidTOX (**Figures 1D–F**) and LipiORDER (**Figures 1J–L**). LipidTOX preferably stains neutral lipid-rich compartments; LipiORDER stains most lipophilic structures but is more sensitive to the biophysical properties of the membrane. We selected LipiORDER as a general membrane staining dye due to its versatility in identifying lipid membranes. After carefully excluding cells with multiple infections, which often contain membrane remnants from dead parasites, the results revealed neutral lipid accumulation near the plasma membranes of the parasites, which is consistent with previous data (Tran et al., 2016). We also identified membranous structures in direct contact with parasite bodies (**Figures 1D–F**, white arrows). These circular membranous structures were approximately 1–4 µm in size (**Figure 1F**). Similar membranous objects structures were detected *via* LipiORDER near the parasite and erythrocyte plasma membranes, some of which appeared to be attached to both the parasite’s and erythrocyte’s plasma membrane (**Figures 1K–L**, white arrows). Collectively, these results indicated the presence of circular membranous structures in proximity to gametocyte bodies in erythrocytes.

**Figure 2:**
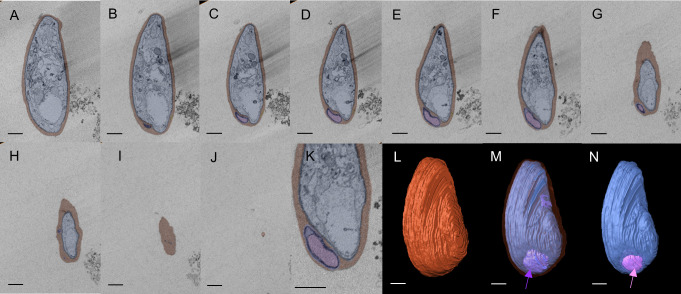
Three-dimensional reconstruction of SBF-SEM imaging showing multi-layered pouch Sections of SBF-SEM images were integrated into a single 3D image (total 38 images). **(A–J)** Series of image sections used to construct the 3D image. Only representative sections are shown. Erythrocytes are shown in red, and exo-membranes are colored purple and pink. **(K)** Enlarged image of **(E)**. **(I)** Erythrocyte membrane is shown in brown. **(M)** Pouches (purple) located between PVM (blue) and erythrocyte membrane (translucent layer). **(N)** Pink segments highlight inner membranous objects within the purple layer as membrane whorl. Scale bar = 1 µm (a-j, l-n), 500nm **(K)**.

**Figure 3:**
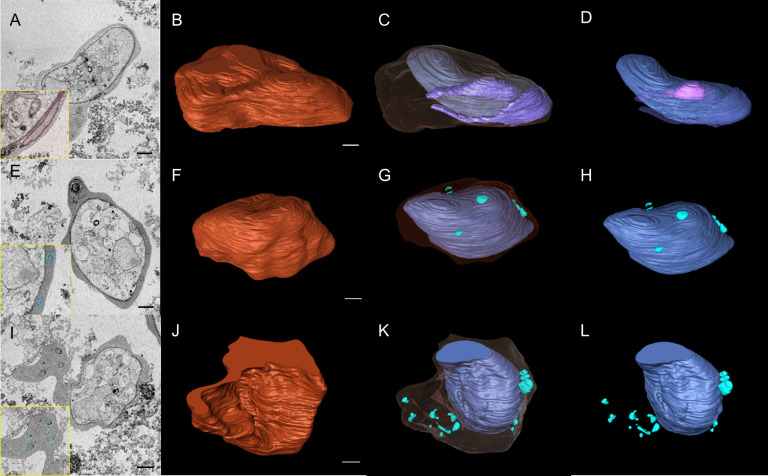
SBF-SEM images revealed complex intracellular membrane organizations in gametocytes. **(A, E, I)** Selected sections of SBF-SEM images with 50 nm intervals. Traces of exo-membranes are shown in insets. **(B, F, J)** 3D images of gametocytes reconstructed from SBF-SEM sections. **(C, G, K)** different size and shape of exo-membranes. The erythrocyte membranes are shown as translucent layer. **(D)** Structure of intracellular parasite and exo-membranes (no erythrocyte membrane is shown), showing that one large membrane includes the smaller membrane (pink). **(H, L)** single-layered, exo-membranes including small membranous objects, globular, spindly, and conjunct membranes exist in cytosol (green). Scale bar = 1 µm.

We then used SBF-SEM to collect sequential cross-sectional images of the gametocytes to reconstruct three-dimensional images of whole gametocytes, and to visualize fine and complex exo-membrane structures. This imaging modality acquires a series of SEM images from the newly exposed sample surfaces after slicing by a diamond knife and constructs three dimensional (3D) structures of cells. An advantage of SBF-SEM compared to other imaging techniques, such as Focused Ion-beam SEM (FIB-SEM), is its ability to acquire structural information from the large area of sample block including multiple cells. SBF-SEM has been used for studying morphological details for malaria parasite-infected erythrocytes (Sakaguchi et al., 2016; Liu et al., 2019; Araki et al., 2020). **Figure 2** shows sequential images of gametocyte segments with inner membranes (a to j) and reconstructed 3D images of the gametocytes showing the balloon-like objects, including the inner membrane (**Figures 2M, N**) with diameter of ~1000–1700 nm (**Supplemental Movie 1**). We also observed different types of exo-membranes with a variety in size that became more evident after 3D reconstruction (**Figure 3**). SBF sections showed the large membranes resided at near the parasitophorous vacuolar membrane (PVM) (expanded sectional view, in yellow squares) (**Figure 3A**, shown as a purple line) and small membranes (blue line) appeared in cytosol of erythrocytes (**Figures 3E, I**). Three-dimensional models revealed large membranes surrounding PVM (**Figures 3C, D**, purple) and small membrane objects scattered in the erythrocyte cytoplasm at near PVM and erythrocyte membrane (**Figures 3G, H, K, L**, green). The size of those small membrane objects ranges over 160 nm – 790 nm.

**Figure 4:**
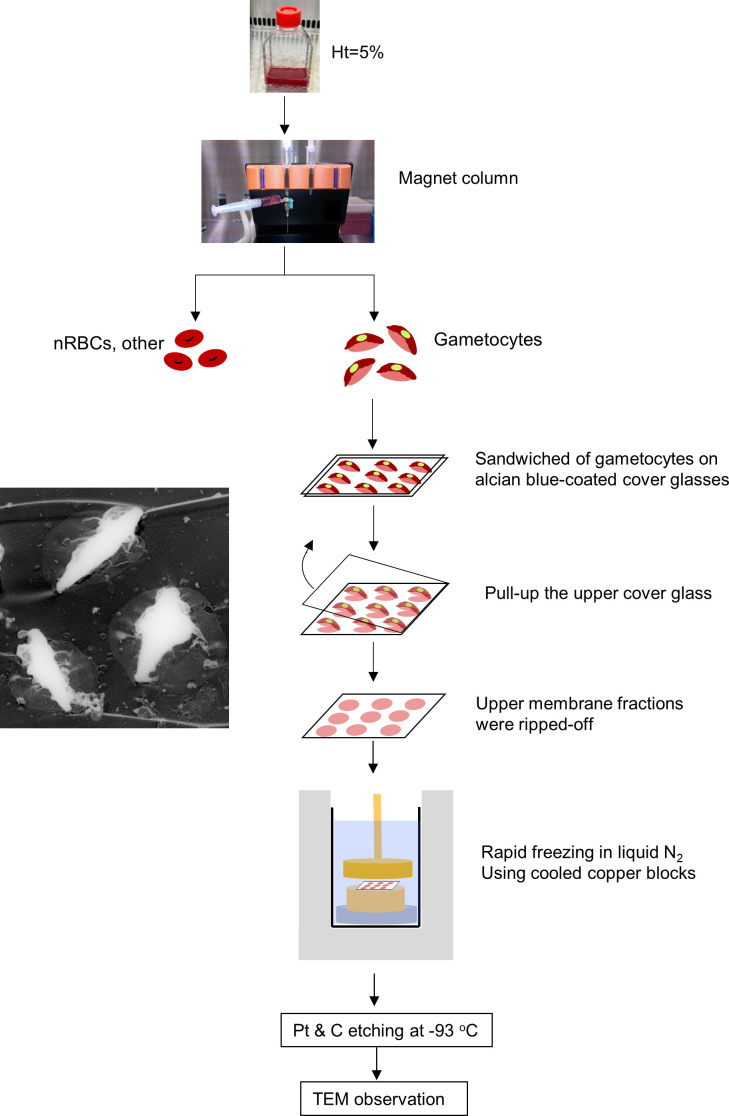
Gametocyte-erythrocyte unroofing sample preparation for TEM observation Flow of unroofing preparation for gametocytes. Gametocytes, after NAG-treatment to remove asexual parasites, were purified slowly using MACS Separators, transferred onto Alcian-blue–treated micro cover glass, and covered with another micro cover glass. The upper membranes of the erythrocytes and gametocytes were removed by pulling up the top micro cover glass. This step removed the upper membrane layers of erythrocytes with gametocytes and exposed the inner structures (TEM image, left panel). After preparing freeze-etching replica with LN_2_ and cooled copper blocks, all replicas were coated with platinum (Pt) and carbon (C) for TEM observation.

**Figure 5:**
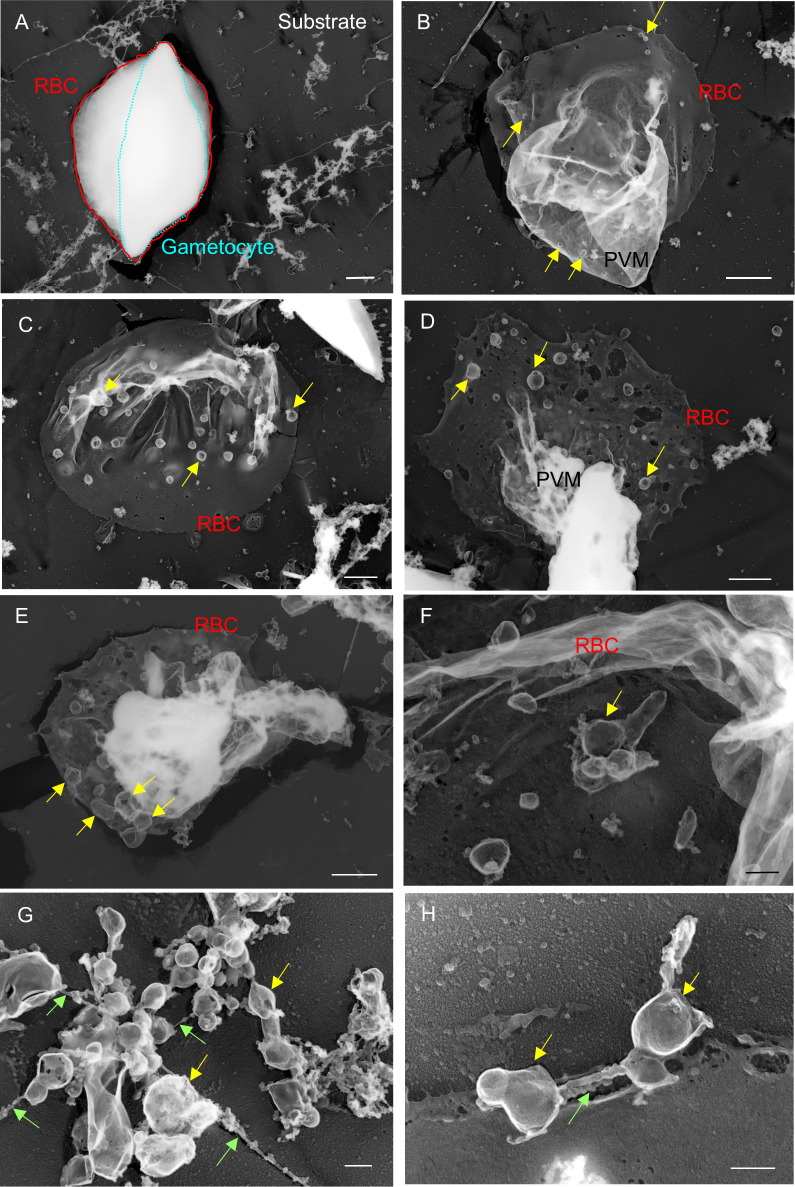
Electron microscopy observations of unroofed gametocytes **(A)** Example of insufficiently unroofed gametocytes. The whole gametocyte structure remained intact after the unroofing process. **(B–H)** Properly unroofed gametocytes. In some cases, gametocyte bodies were also removed by the unroofing process, leaving erythrocyte membranes and associating exo-membranes, depending on the force applied to the cells or variations in the rigidity of individual cells (e.g., c). Bubbles of various sizes appeared on the PVM or interior side of erythrocyte membranes (b–f; yellow arrow). **(G, H)** MC-like objects (yellow arrow) found on erythrocyte membranes. The MC-like objects were bound to each other by tethers (green arrow). These objects were similar in size; however, some were smaller than those found on asexual MCs. Bar = 1 µm **(A–E)**, 200 nm **(F–H)**.

To study the fine structures of the exo-membranes formed in gametocyte-erythrocytes, we exposed the internal spaces of the gametocytes using our established unroofing method (**Figure 4**) and imaged the parasites directly by TEM. Importantly, we excluded the sonication step that was used in our previous study to clean the unroofed samples to preserve the fragile intracellular structures (**Figure 4**). With this soft “pull-up” unroofing method, the parasite, the surrounding exo-membranes, and small structures inside the parasitized erythrocytes remained in the cell, while cytoskeletal structures, which are revealed by the sonication-unroofing method (Hayakawa et al., 2015), appeared relatively flat because they were covered by the cytoplasm contents. **Figure 5A** shows an example of insufficiently unroofed gametocytes (**Figure 5A**, blue dotted line) in which the erythrocytes (**Figure 5A**, red line) remained intact, and no internal structures were exposed. This incomplete unroofing occurs when an insufficiently large shear force is applied to the erythrocyte membrane. With this imaging method, some parts of parasite look whiter than the erythrocyte membrane, PVM, and small bubbles, depending on the thickness of the parasite body. Membrane overlaps also appeared whiter than the thin single-layer membranes. In contrast, successful unroofing removes the outer membrane layers from parasitized erythrocytes attached to the substrate (**Figure 5B**), thus exposing the internal membranes for EM observation. We observed numerous tiny bubble-like structures (**Figure 5B**, yellow arrow; approximately 90–165 nm) directly attached to the PVM and erythrocytes. **Figure 5C** shows numerous bubbles (yellow arrow) with 200–600 nm in diameter covering the inner erythrocyte membrane surface, and these were slightly larger than those shown in **Figure 5B**. Additional bubbles (**Figures 5C–E**, yellow arrow) ranging from 95–670 nm in size were attached to the erythrocyte membrane or PVM. Those size ranges correspond with SBF observations in **Figure 3**. We also observed unique MC-like structures in the gametocytes (**Figures 5F–H**; yellow arrows) (Hayakawa et al., 2015) that were approximately 125–375 nm (**Figure 5F**) and 140–380 nm (**Figure 5H**) in size, and the width of the tethers was ~40 nm (**Figure 5H**, green arrow). These diameters and tether widths were within the range of those found in MCs in our previous study (Hayakawa et al., 2015). Interestingly, we observed multi-linked bubbles with diameters of ~120–460 nm and string widths of ~30 nm (**Figure 5G**, green arrow). These diameters and widths matched those of MCs and MC strings, respectively; thus, we hypothesized that the bubble objects (**Figures 5B–H**) were MCs or remnants of MCs present in the gametocyte stage.

**Figure 6:**
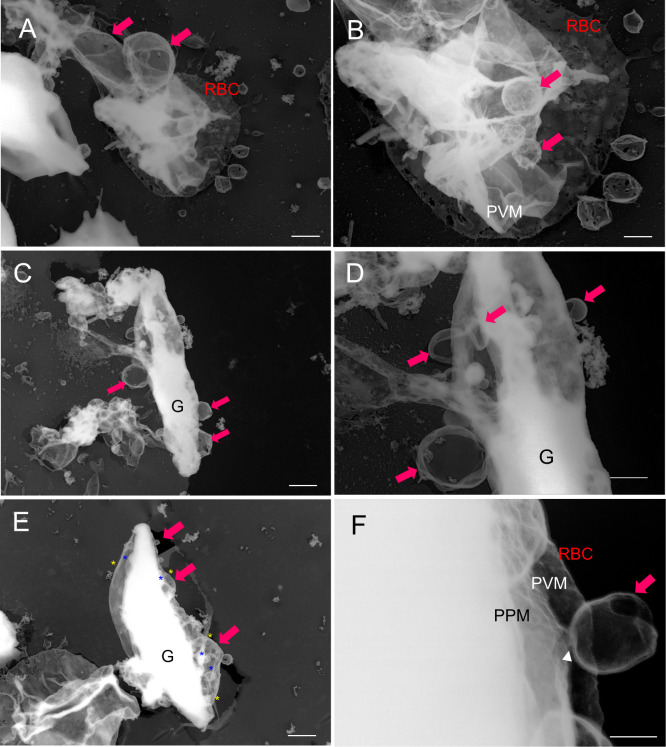
Budding pouches were revealed *via* TEM analysis of unroofed structures **(A–F)** Large membrane (“pouch”) budding from the PVM or parasite body (pink arrow). **(D)** The membrane whorls (that were also observed in b, and e) are marked by yellow asterisks (outer membranes) and blue asterisks (inner membranes). **(F)** An enlarged view of d. TEM, transmission electron microscopy; PVM, parasitophorous vacuole membrane; PPM, parasite plasma membrane; G, gametocytes. Bar = 1 µm **(A, C, E),** 500 nm **(B, D)**, 100 nm **(F)**.

**Figure 7:**
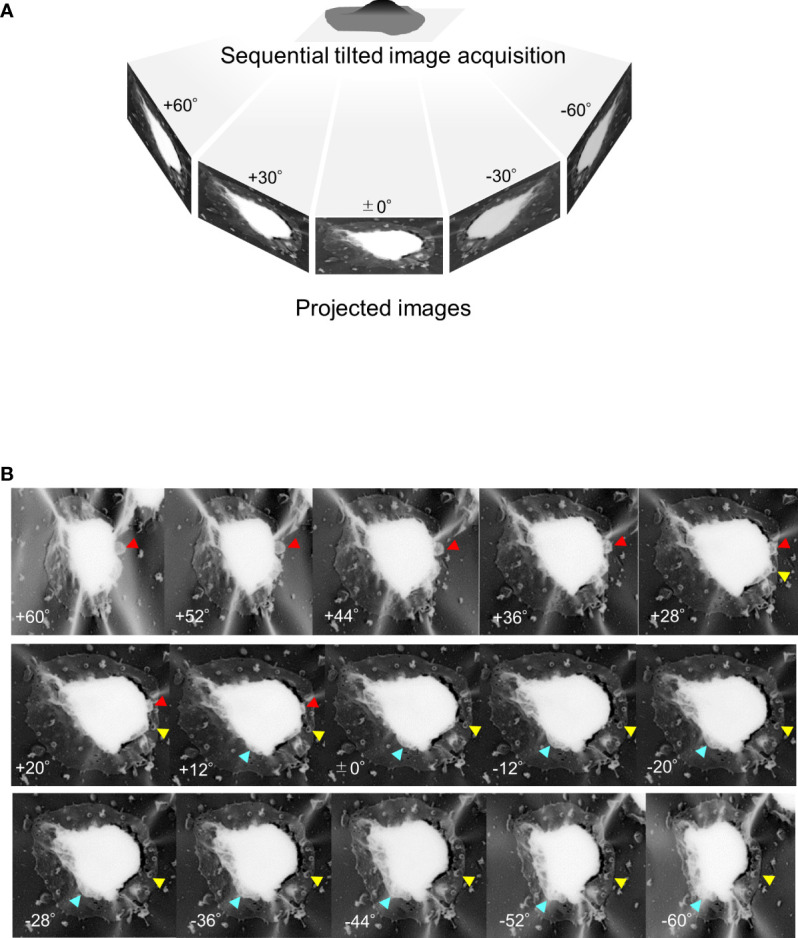
Tilting images of unroofed gametocyte-erythrocytes **(A)** Sequential tilting images of unroofed specimens were acquired with tilting steps at every 2° from −60° to +60°. **(B)** Representative images of unroofing-tilt images. By observing the same sample from different angles, the positional relationships between the small bubbles and the host erythrocyte membrane were clearly seen; for example, binding of the small bubble to the erythrocyte membrane (yellow arrowhead) or the gametocyte body (red arrowhead). Also, large exo-membrane; pouches of a kangaroo are showed (blue arrowhead).

In later-stage gametocytes, we observed membranous objects of different shapes, sizes, and textures that resembled “air balloons” or “kangaroo pouches” (**Figure 6**). **Figure 6A** illustrates two air-balloon-like objects with diameter of ~730–1980 nm (pink solid arrow) and globular balloons with diameter of ~500–700 nm. Other membranous objects attached to mature stage gametocytes (**Figures 6C, D**, ~230–960 nm) and balloon-shape objects emerging from the PVM (**Figure 6E**, ~100–1320 nm) were found. Interestingly, some small balloons has formed inside the large balloons, resulting in structures resembling membrane whorls (**Figures 6B, D, E**), and we marked asterisk (*) in (d) as representative membranes. There were also balloon-like objects that had directly budded from the PVM (**Figure 6F**, white arrowhead). Overall, the size of these balloons ranged from ~100 nm to –1980 nm, which is compatible with the size of MCs. For better spatial visualization, we imaged unroofing specimen by tilting from −60° to +60° (**Figure 7A**) to clarify the positional relationships between the bubble-like structures, PVM, and erythrocyte membrane (**Figure 7B**, **Supplemental movie 2**). These images clearly show that the small bubbles are connected to the PVM (red arrowheads) and the cytoplasmic side of the erythrocyte membranes (yellow arrowheads) (**Figure 7B**), providing additional details on the exo-membrane structures revealed in the unroofing images (**Figure 5**, **6**). These data also complement the information on the gametocyte-erythrocytes inner structures shown by the fluorescence and SBI-SEM data (**Figures 1**–**3**).

## Discussion

Our direct intracellular observations using unroofing-TEM and SBF-SEM provided new insights into the stereoscopic exo-membranes in gametocyte-infected erythrocytes. The first type of membrane forms relatively small bubbles, similar to MCs, that are either with or without tethers/strings. The MC-like small bubbles are smaller in both volume and dimensions than typical MCs seen during the asexual stages (Hanssen et al., 2008; Cyrklaff et al., 2011; Hayakawa et al., 2015) (Hayakawa et al., 2015). In addition, the bubbles were associated with other bubbles and occasionally overlapped without tethers or strings (**Figures 5C–H**). Our data did not identify typical MCs, which are distinguishable from other membranous structures based solely on appearance; however, the bubbles had similar structural characteristics, with spherical or “baroque pearl”-shaped asexual-MCs and small blebs on the surface of some bubbles (Hayakawa et al., 2015). Additionally, there were often small bubbles connected to the erythrocyte membrane skeleton, as seen in asexual MCs (Hayakawa et al., 2015). The second type of exo-membranes resembled balloons or the pouches of a kangaroo (**Figure 6**) (**Supplemental Movie 3**) and similar pouched shapes were also observed in our fluorescence imaging data (**Figure 1**). Based on the rise-out position and localization pattern of the pouches, these pouches resembled the sexual-stage tubular intraerythrocytic compartments (STICs) (Eksi et al., 2002) and unique globular exo-membranes shown in a report by Kass et al. (1971). The bubbles or large pouches were observed in approximately 50 successfully unroofed cells, and approximately 30 pouches and 30 bubbles were found in total. Within the number of cells observed in this study, some variations in appearance of membranes, although they are not critical, were noticed probably due to the different physical impact of unroofing conditions to each stage of gametocytes.

Many researchers have identified or, at least, found evidence suggesting the development of additional membranes in asexual to gametocyte-erythrocytes (summarized in **Table 1**). The data quality of these membrane structural details have been improved with the development and increased resolution of imaging modalities. Early examples of features clarified by improvements in imaging are the Garnham bodies in gametocytes in 1933 (Garnham, 1933) and the morphological development of parasites by Giemsa staining images. These reports were followed by electron microscopy studies from the 1970s to the 1980s that identified exo-membranes that were named as membrane whorls, a tubovesicular network (TVN), and MCs in parasitized erythrocytes (Ponnudurai et al., 1986). Although the original descriptions of the Garnham body did not define its identity or function, some studies reported globular objects in gametocytes (in both the early and mature stages) that formed lipid membrane whorls that occasionally contained hemozoin *in vitro* (Orjih, 2012), and membrane whorls often appeared in patients’ blood samples as the identifying characteristic of the Garnham bodies (Mohanty et al., 2019). Considering the characteristics of these unique membrane structures, what we have observed in this study seem to share morphological features with Garnham bodies.

**Table 1:** Summary description of “pouches” and other structures in RBCs infected with *P. falciparum*.

	Maurer’s clefts	Pouches	TVN	Circular vesicles or STIC
Size of bodies	100 –650 nm	300 –2200 nm	indeterminate form	indeterminate form
Shapes	globular and oval with strings and tethers	globular, balloon	extended membrane, various	circular
Localizations	cytosol and inside RBC membrane	cytosol of RBC, budding from PVM and parasites	extension from PVM	budding from PVM
Asexual or gametocyte	asexual and gametocyte	gametocyte	asexual	asexual
Protein transport	+	?	+	?
Cholesterol/lipid transport	+	?	+	?

Regarding exo-membrane functionality, studies have shown that antibodies such as Pfs230 (Eksi et al., 2002), PfSBP1 (Petter et al., 2008), Pf13_0006 (Mwakalinga et al., 2012), and ring exported protein-1 (Dearnley et al., 2016) labelled features in gametocytes, suggesting that transportation systems in the host erythrocyte cytoplasm, including MC-like or MC-remnant structures, likely exist in gametocytes. However, Eksi et al. also observed different membrane objects, i.e., STICs, in gametocyte-erythrocytes (Eksi et al., 2002) that could not be detected by specific markers for MCs. There are still many unknowns regarding the transition to sexual gametocytes, but our focus in this study was to obtain stereoscopic structures of exo-membranes in gametocyte-erythrocytes using the unroofing technique, providing unique structural data and information for future cell biological studies. The exact identification of each gametocyte-specific exo-membrane system and its function will require more systematic studies involving a combination of genetic modifications of well-known marker proteins and cutting-edge imaging modalities.

## Data availability statement

The original contributions presented in the study are included in the article/**Supplementary Material**. Further inquiries can be directed to the corresponding authors.

## Ethics statement

Human erythrocytes, obtained from O+ blood, were procured from the Japanese Red Cross Society (authorization number: 25J0045, 28J0058). Blood donor information was not available. Malaria parasite culture in human erythrocytes for clinical research was approved by the Bioethics Committee for Epidemiologic Research, Jichi Medical University (#18-011), and by the Ethics Committees of the Nagasaki University (#191226226). Written informed consent for participation was not required for this study in accordance with the national legislation and the institutional requirements.

## Author contributions

EH conceptualized the design and acquired funding for this study. FT performed fluorescence microscopy imaging. EH and JU performed unroofing-TEM imaging. MW and EH performed tilting-TEM, and the results were analyzed by MW. NO, MM, and EH performed SBF-SEM observation. NO and MM analyzed SBF-SEM. EH wrote the original manuscript. EH and FT edited the manuscript. All authors validated the data and reviewed the manuscript.

## Funding

This work was partially supported by grants given by JSPS KAKENHI (grant numbers 18K11135 and 22K11834) to ESHH, and Cooperative Study Programs of National Institute for Physiological Sciences to ESHH.

## Acknowledgments

We thank Dr. Shinya Miyazaki, Nagasaki University for providing a high-quality gametocyte culture. We also thank Dr. Thomas Wellems, Laboratory of Malaria and Vector Research, National Institute of Allergy and Infectious Diseases, National Institutes of Health, for the advice and encouragement. The authors are grateful to Atsuko Imai, Nobuko Hattori (National Institute for Physiological Sciences), and Tom Kouki (Jichi Medical University) for their helpful technical assistance. We thank Suzanne Leech, Ph.D., from Edanz (https://jp.edanz.com/ac) for editing a draft of this manuscript.

## Conflict of interest

MW is employed by Hitachi High-Tech Corporation Solution. This study did not receive funding from Hitachi High-Tech Corporation Solution. FT (The Department of Cellular Architecture Studies, Institute of Tropical Medicine, Nagasaki University), is supported by Shionogi & Co., Ltd. The funder was not involved in the study design, collection, analysis, interpretation of data, the writing of this article or the decision to submit it for publication.

The remaining authors declare that the research was conducted in the absence of any commercial or financial relationships that could be construed as a potential conflict of interest.

## Publisher’s note

All claims expressed in this article are solely those of the authors and do not necessarily represent those of their affiliated organizations, or those of the publisher, the editors and the reviewers. Any product that may be evaluated in this article, or claim that may be made by its manufacturer, is not guaranteed or endorsed by the publisher.
