# Supplementary figures and images for: Budding pouches and associated bubbles: 3D visualization of exo-membrane structures in plasmodium falciparum gametocytes
Source: Front Cell Infect Microbiol. 2022 Aug 22;12:962495. doi: 10.3389/fcimb.2022.962495 (PMC9441640; doi:10.3389/fcimb.2022.962495)

## Slide 1
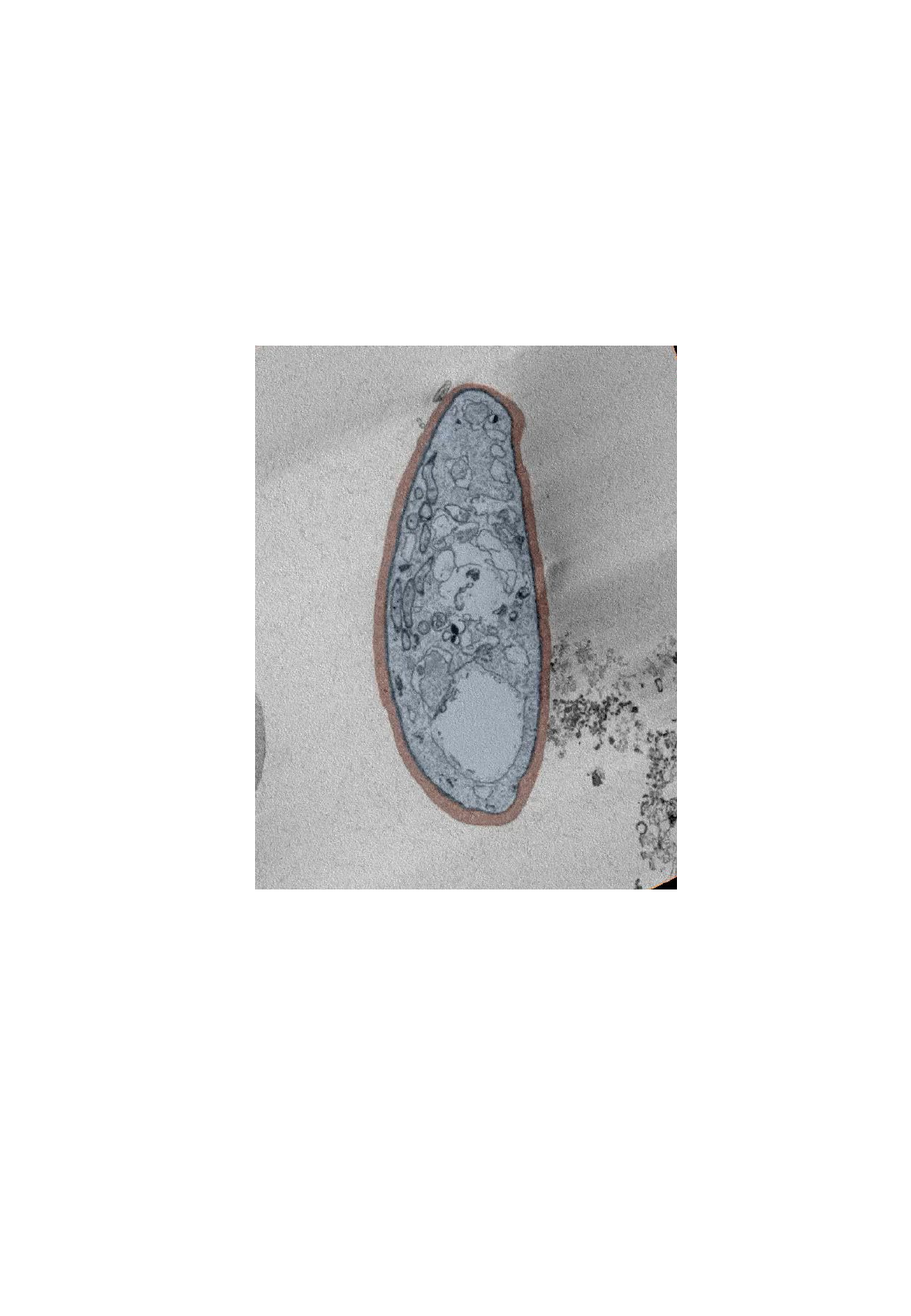

Supplement: Supplementary file 1 [file Presentation_1.pptx]

## Slide 1
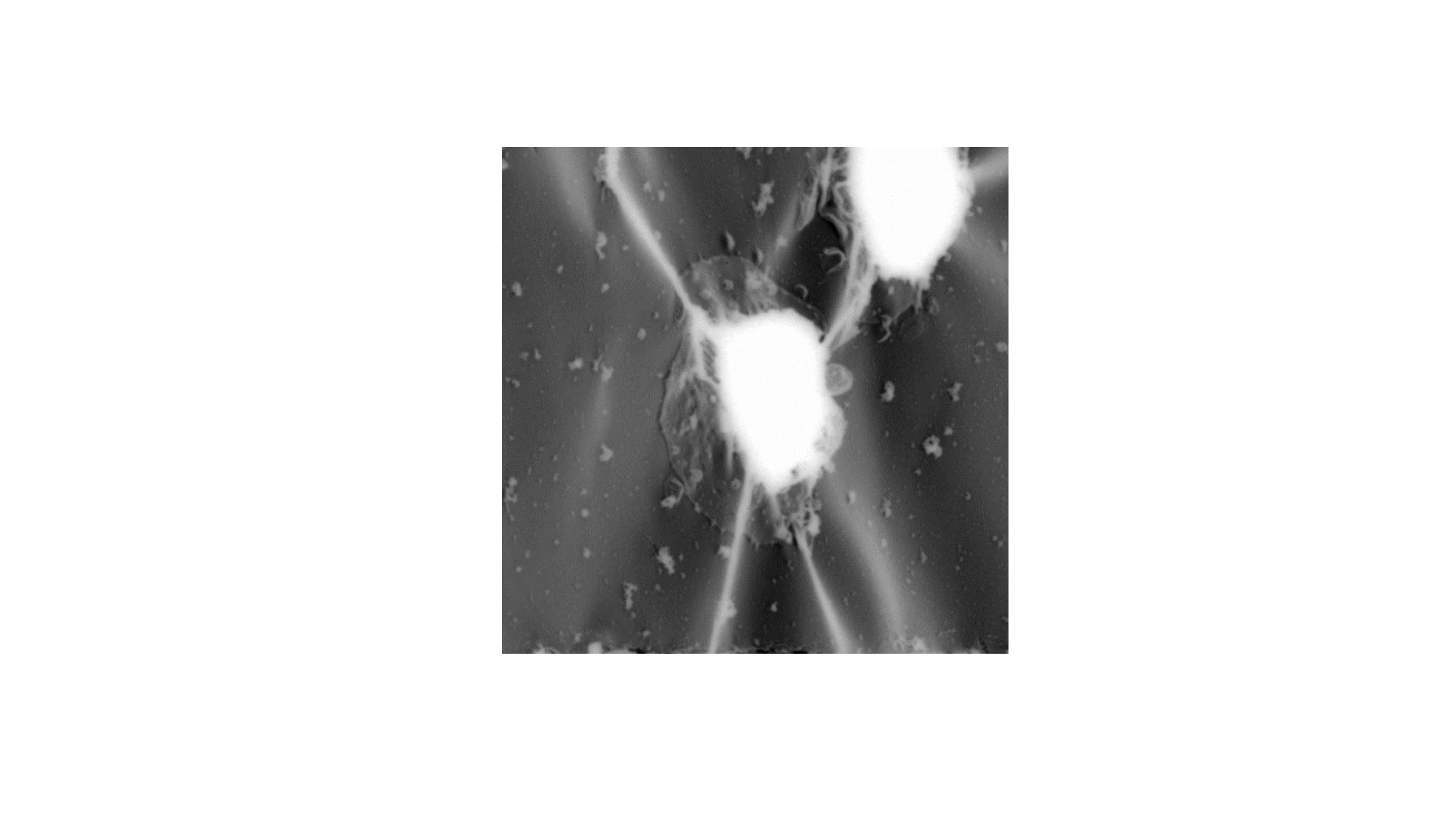

Supplement: Supplementary file 2 [file Presentation_2.pptx]

## Slide 1
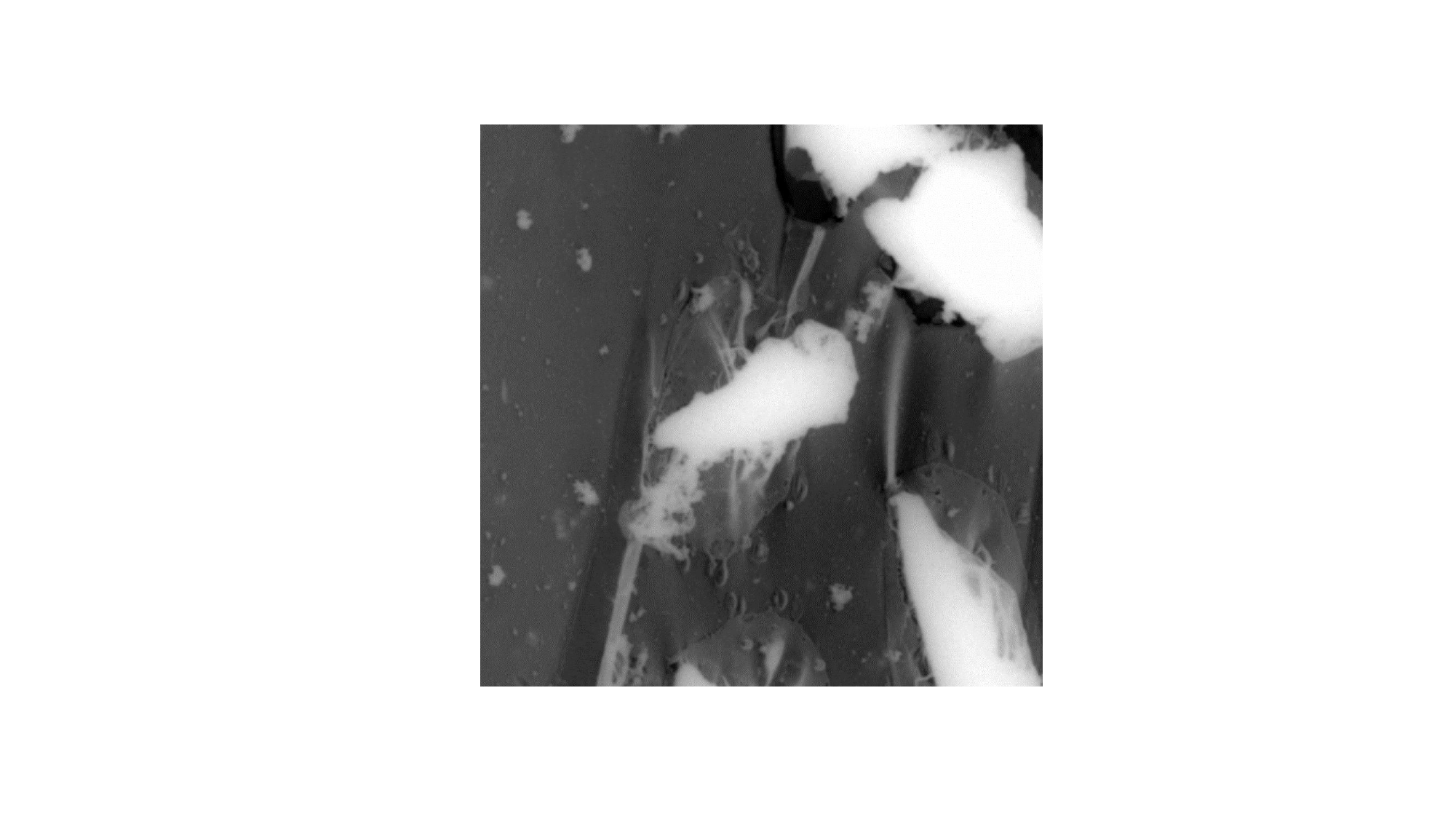

Supplement: Supplementary file 3 [file Presentation_3.pptx]
