# Supplementary material for: Budding pouches and associated bubbles: 3D visualization of exo-membrane structures in plasmodium falciparum gametocytes
Source: Front Cell Infect Microbiol. 2022 Aug 22;12:962495. doi: 10.3389/fcimb.2022.962495 (PMC9441640; doi:10.3389/fcimb.2022.962495)
Supplement: Supplementary file 4 [file Table_1.docx]

**Supplementary Movie 1**

Sequential 3D re-construction of SBF-SEM images. The 3D structure of late-stage gametocytes determined using SBF-SEM sections. This supplemental movie corresponds to Figure 2.

**Supplementary Movie 2**

Continuous tilting images with aligned and inverted contrast. This movie corresponds to Figure 7.

**Supplementary Movie 3**

Sequential tilting images of late-stage gametocyte-erythrocytes with aligned and inverted contrast. Parasite stage and morphology in this movie are relevant to Figure 6.
